# Brain is an endocrine organ through secretion and nuclear transfer of parathymosin

**DOI:** 10.26508/lsa.202000917

**Published:** 2020-10-21

**Authors:** Bin Yu, Yizhe Tang, Dongsheng Cai

**Affiliations:** 1 Department of Molecular Pharmacology, Albert Einstein College of Medicine, Bronx, NY, USA; 2 Institute for Aging Research, Albert Einstein College of Medicine, Bronx, NY, USA; 3 Diabetes Research Center, Albert Einstein College of Medicine, Bronx, NY, USA

## Abstract

This study reports that parathymosin (PTMS) is secreted by hypothalamic stem/progenitor cells (htNSC) to inhibit senescence of recipient cells such as fibroblasts. Upon release, PTMS is rapidly transferred into the nuclei of various cell types, including neuronal GT1-7 cells and different peripheral cells, and it is effectively transferred into neuronal nuclei in various brain regions in vivo. Notably, brain neurons also produce and release PTMS, and because neuronal populations are large, they are important for maintaining PTMS in the cerebrospinal fluid which is further transferable into the blood. Compared with several other brain regions, the hypothalamus is stronger for long-distance PTMS transfer, supporting a key hypothalamic role in this function. In physiology, aging is associated with declines in PTMS production and transfer in the brain, and *ptms* knockdown in the hypothalamus versus hippocampus were studied showing different contributions to neurobehavioral physiology. In conclusion, the brain is an endocrine organ through secretion and nuclear transfer of PTMS, and the hypothalamus–brain orchestration of this function is protective in physiology and counteractive against aging-related disorders.

## Abstract (precis)

The hypothalamus and brain neurons orchestrate the secretion and nuclear transfer of PTMS which is neuroprotective against aging-related disorders.

## Introduction

The nervous system plays a leading role in controlling whole-body physiology and pathophysiology, including aging-associated physiological declines. In mammals, different brain regions should have specific contributions to aging, for instance, the hippocampus has long been known for aging-related cognitive disorders, and we recently appreciated that the hypothalamus is important for the endocrine control of whole-body aging (Satoh et al, 2013; Zhang et al, 2013), although the underlying cell mechanism involves hypothalamic neural stem/progenitor cells (htNSC) (Zhang et al, 2017). Because protein or peptide secretion is a key feature of endocrine function, this project was designed to examine protein or peptide secretome of htNSC, and we found that these cells importantly secrete parathymosin (PTMS). PTMS is a protein which has been poorly understood except for its sequence and limited biochemical information in the literature. This protein was initially isolated from rat thymus and found to have 43% structural identity with thymosin, thus leading to this name (Haritos et al, 1985; Komiyama et al, 1986; Trompeter & Söling, 1992), and later it was purified from liver and was also given a different name (Okamoto & Isohashi, 2000). Based on its mRNA sequence, PTMS is highly conserved across species and is a small zinc-binding acidic protein containing 101–amino acid residues. Besides, PTMS was known to have an operative bipartite nuclear-localization signal which could interact with histone (Kondili et al, 1996; Trompeter et al, 1996). PTMS appears to be widely expressed in the body, much more than what the name suggested, and it was already clear that the thymus is not a key site for PTMS expression (Clinton et al, 1989; Okamoto & Isohashi, 2000). On the other hand, very little is known about its function, and it is completely unknown if it is relevant to the brain, neurons, or hypothalamus. In this work, we discovered that PTMS is a brain-secretory protein which is transferable among neuronal nuclei, suggesting that brain is an endocrine organ through secretion and nuclear transfer of PTMS with a neuroprotective role in physiology.

## Results

### The secretome of htNSC inhibits cell senescence in a co-culture model

**Figure 1.**
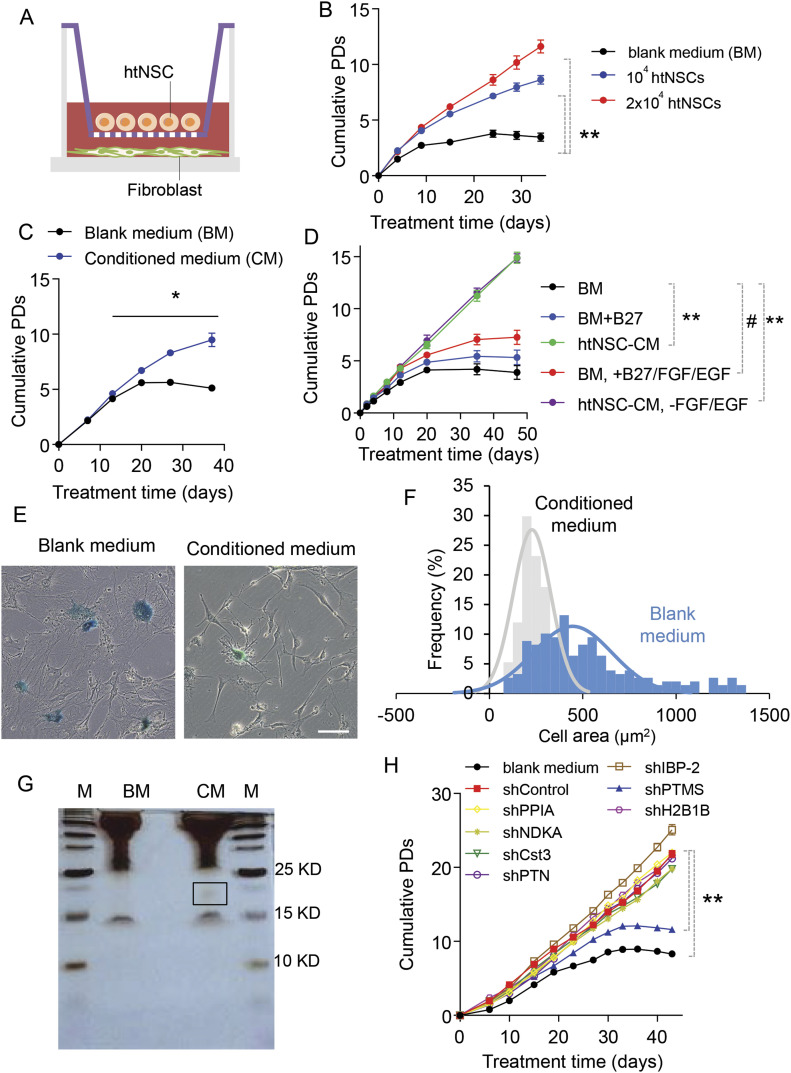
Identification of anti-senescence nuclear peptide PTMS released by htNSC. **(A)** Schematic diagram depicting htNSC-primary fibroblast co-culture model. **(B)** Growth kinetics of primary mouse skin fibroblast co-cultured with indicated numbers of htNSC or with blank htNSC medium (labelled as “blank medium [BM]”). Cumulative Population Doublings (PDs) of fibroblasts were measured at each passage until the PD in BM group began to decrease. **P < 0.01 (ANOVA/post-hoc), compared between indicated groups across time points from day 9 to 35, n = 3 each group per time point. **(C, D)** Growth kinetics (PDs) of mouse skin fibroblasts co-cultured with BM or htNSC-conditioned medium (labelled as “CM”) (C), or BM with B27, B27 plus bFGF, and EGF (10 ng/ml each) **(D)**. *P < 0.05 from day 13 to 37, #*P* < 0.05 from day 35 to 47, **P < 0.01 from day 20 to 47, compared with time-matched groups indicated by solid lines (ANOVA/post hoc), n = 3 each group per time point. **(E, F)** Senescence morphology of continuously passaged fibroblasts cultured with blank or htNSC-CM. **(E, F)** Cellular senescence displayed at passage 6 as revealed by SA-β-Gal staining (E) and cell size profiling (F). Scale bar, 100 μm. Data represented n = 134 cells for blank group and n = 163 for CM group. **(G)** Representative image of silver-staining of SDS–PAGE separating peptides of htNSC-CM which showed anti-senescence effect, whereas BM was included to provide a baseline control. A smear area (indicated by black square) in the lane of htNSC-CM was analyzed by mass spectrometry for peptide identification. Molecular marker ladder (labelled as “M”) was included on both sides. **(H)** Growth kinetics (PDs) of fibroblasts co-cultured with individual htNSC cell line expressing non-target shRNA control or specific shRNA targeting each of the mass spectrometry-identified peptides. Culture with BM was also included a background control, **P < 0.01 (ANOVA/post-hoc) compared between indicated groups across time points from day 15 to 43, n = 4 each group per time point. All values represent the mean ± SEM.

**Figure S1.**
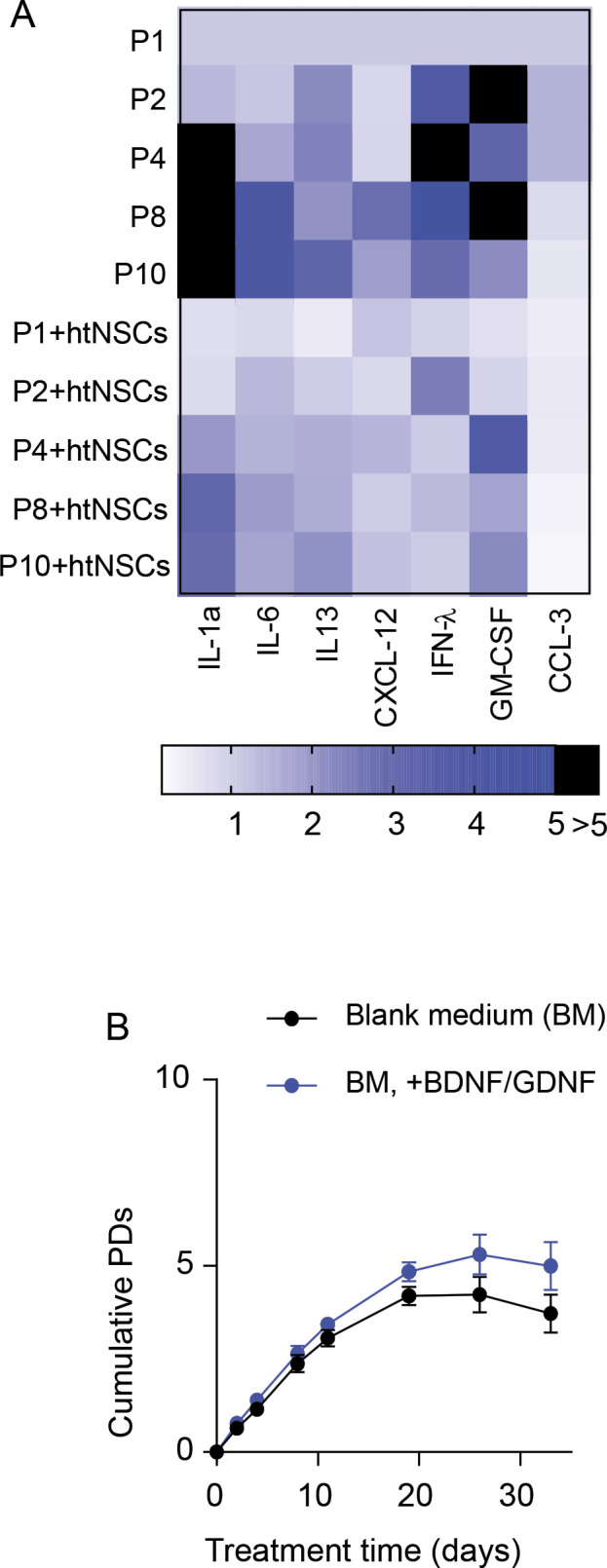
Reduced fibroblast cell senescence due to htNSC secretion. **(A)** Heat map representing mRNA levels of senescence-associated secretory phenotype molecular markers (columns) of fibroblasts at indicated passages (P1-P10) with htNSCs co-culture or blank medium control. Heat map key indicates fold changes normalized to that of P1 fibroblasts. Values represent the average of three different measurements, and n = 4 biological samples per group were used. IL, Interleukin; CXCL-12, C-X-C Motif Chemokine Ligand 12; IFN, interferon; CCL-3, Chemokine (C–C motif) ligand 3. **(B)** Growth kinetics (Population Doublings) of primary mouse skin fibroblasts co-cultured with blank medium with or without brain-derived neurotrophic factor and GDNF (10 ng/ml each). ANOVA/post-hoc analysis was applied, n = 3 each group per time point. Values represent the mean ± SEM.

Cellular aging is characterized with replicative senescence by which cells cease to divide (Kuilman et al, 2010; López-Otín et al, 2013). To initiate the investigation of peptide and protein secretome of htNSC in affecting aging process, we started with a screening experiment to identify if there exists a peptide or protein released by htNSC to reduce senescence of primary fibroblasts. As shown in Fig 1A, co-culture was set up using a transwell system with mouse skin fibroblasts (MSFs) in the lower compartment and htNSC spheres in the upper compartment, then we measured growth kinetics of fibroblasts at each passage. After 2 wk of cell passaging, growth of fibroblasts reached a plateau stage as these cells stopped increasing the cumulative average population (Fig 1B), indicating that most cells became replicatively senescent. Co-culture with htNSC dramatically rescued the replicative senescence of fibroblasts in a dose-dependent manner, and a significant difference was observed after day 10 (Fig 1B). Then, to assess if this effect was mediated by any secreted factors, we obtained htNSC-conditioned medium and applied to fibroblasts. As the results showed in Fig 1C, htNSC-conditional medium greatly protected against replicative senescence of fibroblasts. Considering that the standard medium of htNSC culture contained additional ingredients (B27 supplements, EGF and bFGF) compared with the culture medium of fibroblasts, we further experimentally address this difference. Fibroblast replicative aging remained when these ingredients were added to its culture medium (Fig 1D), and on the other hand, the anti-aging effect of htNSC-conditioned medium remained intact even if EGF and bFGF were removed (Fig 1D). Besides proliferative kinetic, we analyzed senescence-related β-galactosidase (SA-β-gal) staining, cell morphology, and senescence-associated secretory phenotype. As shown in Fig 1E and F, senescent fibroblasts were SA-β-gal positive, which became flattened and shape-enlarged, whereas co-cultured with htNSC significantly decreased SA-β-gal positive ratio and shifted the cell size frequency curve to the left. The mRNA of senescence-associated secretory phenotype cytokines of fibroblasts accumulated since passage 4, but it remained low at passage 10 when co-cultured with htNSC (Fig S1A). To summarize, htNSC secrete certain factor(s) to prevent against cellular replicative aging.

### PTMS from htNSC secretome is anti-senescent

**Figure S2.**
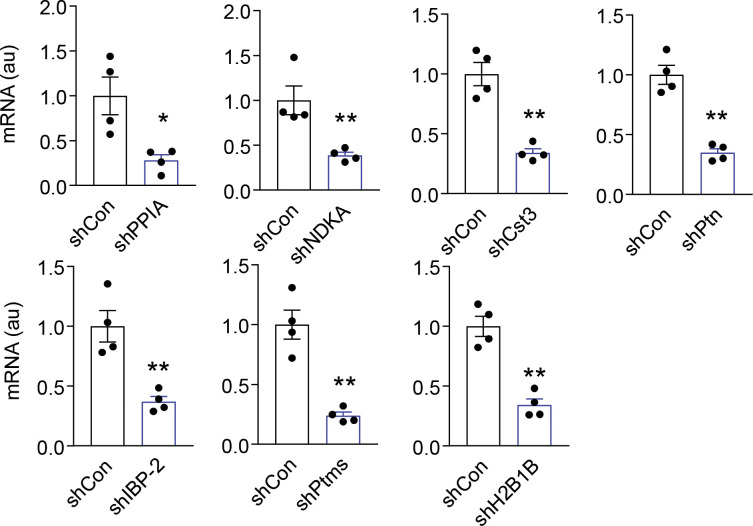
Knockdown of peptides that are potentially secreted by htNSCs. Knockdown of individual candidate genes of htNSCs by individual lentiviral shRNAs compared with scramble control shRNA. *P < 0.05; **P < 0.01 (two-tailed unpaired *t* test), n = 4 biological samples per group; values represent the mean ± SEM.

In this context of the above findings based on co-culture and conditioned medium, we asked if any known neurotrophic factors could be accountable. We targeted a few prototypical ones, including brain-derived neurotrophic factor and glial cell line-derived neurotrophic factor (GDNF). In experiments, brain-derived neurotrophic factor and GDNF (10 ng/ml each) were added to fibroblast culture medium, but we found that they did not lead to any rescue of fibroblast replicative aging (Fig S1B). To unveil the potential anti-aging peptides or proteins released by htNSC, we subjected htNSC-conditioned medium to gel identification with silver staining and observed the presence of a smeared protein band between 15 and 20 kD in htNSC-conditioned medium (Fig 1G). Thus, we isolated this area from the gel and analyzed the sequences of peptides in this area via nanoLC-MS/MS analysis. The most abundant candidate proteins or peptides revealed by this method included PPIA, NDKA, Cst3, PTN, IBP-2, Ube2v1, PTMS, H2B1B, and TI-225. Surprisingly, only Cst3, PTN, and IBP-2 were known as secretory factors, whereas others are either poorly characterized (such as PTMS and TI-225) or usually do not represent secretory proteins (such as PPIA, NADK, Ube2v1, and H2B1B). Based on our recent finding which revealed that htNSC have an important function of abundantly releasing exosomes (Zhang et al, 2017), it is mostly likely that some of these proteins could be released through extracellular vesicles. We generated individual knockdown htNSC cell lines through lentivirus-harboring shRNA against each of these identified peptides or proteins. Through qRT-PCR, we confirmed that several htNSC lines developed from this strategy were comparable in terms of knockdown efficiency (Fig S2). We then co-cultured fibroblasts with each of these knockdown htNSC cell lines. The results revealed that PTMS knockdown dramatically abolished the anti-aging effect of htNSC co-culture, whereas none of the other knockdowns did so (Fig 1H). We thus analyzed the literature and recognized that PTMS has been poorly characterized except that it is known as a small zinc-binding acidic protein which is conserved across species, and it contains an operative bipartite nuclear-localization signal and was found to interact with histone (Kondili et al, 1996; Trompeter et al, 1996). Hence, in the context of our results, we decided to study the possible endocrine feature of this protein.

### PTMS secretion from htNSC and its rapid nuclear transfer in vitro

**Figure S3.**
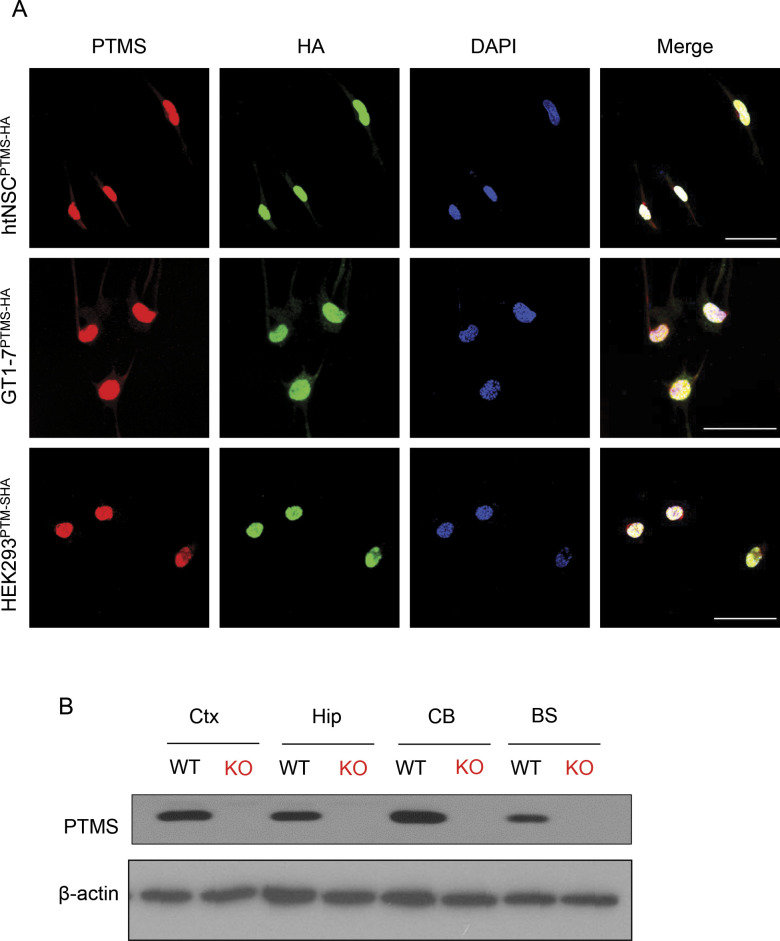
Validation of PTMS antibody. **(A)** Different cell types, including htNSCs, GT1-7, and HEK293 cells were infected via lentiviral HA-tagged PTMS (PTMS-HA) and established as stable cell lines. These cells were analyzed using co-immunostaining of PTMS and HA using anti-PTMS antibody and anti-HA antibody to reveal co-localization of these two staining signals. DAPI nuclear staining was also included. Scale bar, 50 μm. **(B)** Cortex (Ctx), hippocampus (hip), cerebellum (CB), brain stem (BS) from Ptms knockout (KO) mice, and wild-type (WT) controls were subjected to Western blot using PTMS antibody. Blot with β-actin antibody was used as an internal control.

**Figure 2.**
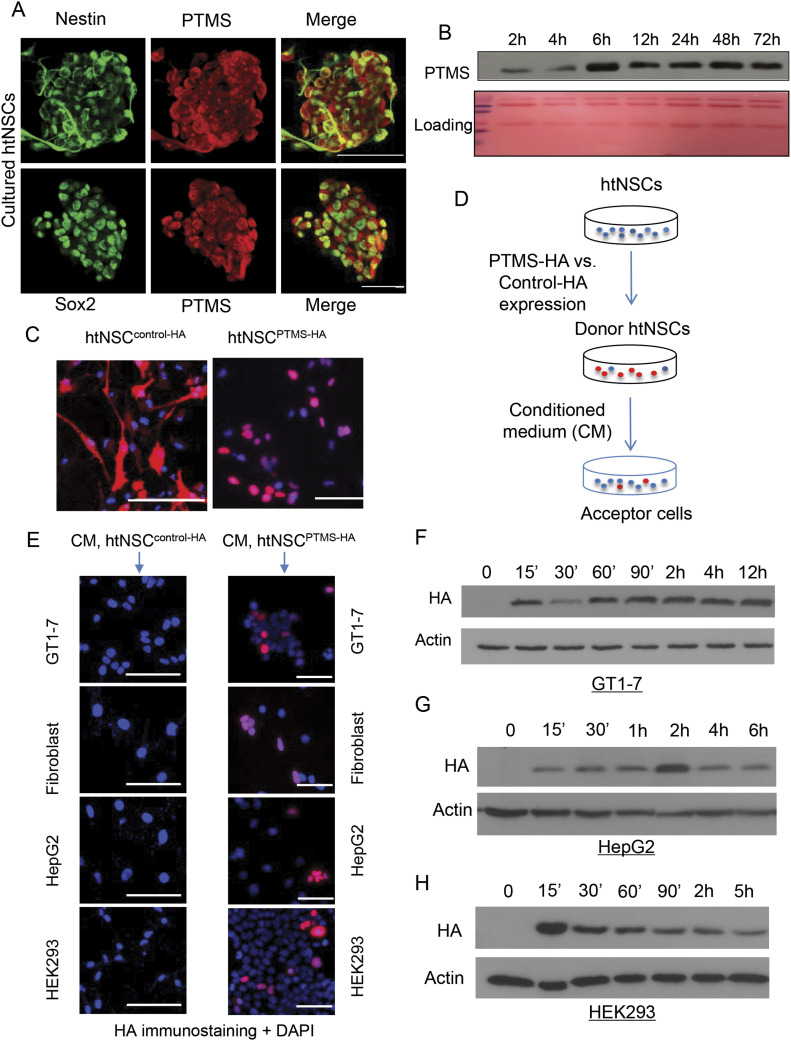
Protein expression, secretion and transfer of PTMS by htNSC in vitro. **(A)** Representative sections of htNSC spheres were double immunostained for PTMS and neural stem cell marker Nestin or Sox2. Scale bar, 50 μm. **(B)** htNSC-conditioned medium (CM) was collected in a time course of 72 h and was detected for PTMS levels by immunoprecipitation—Western blot. Ponceau staining was used as a loading control. **(C)** htNSC were infected with the lentiviruses expressing HA-tagged PTMS (PTMS-HA) or HA-tagged scramble control (Control-HA) driven by CMV promoter. **(D, E, F, G, H)** CM of donor htNSC expressing PTMS-HA (labelled as htNSC^PTMS-HA^) versus Control-HA (labelled as htNSC^control-HA^) was added to various acceptor cells for various of time, as elucidated in (D). **(E)** Subcellular localization of HA-tagged protein in acceptor cells was detected by HA immunostaining after 24 h CM incubation (E). DAPI nuclear staining indicated all cells in the fields. Scale bar, 100 μm. **(F, G, H)** GT1-7 (F), HepG2 (G), and HEK293 (H) cells treated with CM of htNSC^PTMSl-HA^ were collected at the indicated time periods of incubation, thoroughly washed, and lysed for HA Western blot. Western blot did not reveal any HA signal in the above cells incubated with htNSC^control-HA^ CM.

A suitable PTMS antibody should be necessary for studying its production and secretion in mouse models, although commercially there are a few PTMS antibodies against human species, we tested them for mouse samples but none of them turned out to be workable. Thus, we decided to generate a suitable antibody against this protein of mouse origin. We screened several versions of customized rabbit antibodies against different N-terminal sequences of mouse PTMS and established an antibody, which was validated to be specific, as it specifically recognized an exogenously expressed PTMS but did not lead to a signal for various samples from *ptms* knockout mice (Fig S3). Using this antibody, we performed immunostaining for htNSC spheres and confirmed that htNSC indeed abundantly contained PTMS which largely co-localized with NSC markers Sox2 and nestin (Fig 2A). Furthermore, we profiled the kinetics of PTMS secretion by htNSC. As shown in Fig 2B, PTMS secretion from htNSC was quickly detectable within 2 h into serum-free medium and reached a steady state at 6 h, indicating that PTMS secretion by htNSC is fast. We were interested in studying the property of PTMS release independent of its gene production, and thus we generated an in vitro htNSC model with exogenous expression of HA-tagged PTMS. Because the N-terminal could be important for mediating its release, we added HA tag to the C-terminal of PTMS protein. Various controls were designed, including the size- and sequence-matched control based on using scrambled cDNA sequence of PTMS, and HA was also tagged at the C-terminal. Indeed, both were expressed in htNSC according to HA staining, but notably PTMS-HA was present mainly in the nucleus, whereas Control-HA was also widely present in the cytoplasm (Fig 2C), which further suggested that PTMS can work in the nucleus. Also, through analyzing HA in culture medium, we confirmed that PTMS-HA was abundantly released, whereas control-HA did not lead to any level of release. Subsequently, we studied how PTMS from secretion could act on target cells, for example, whether it act on cell membrane as similarly as many other peptidyl hormones do, or if it could be transferred into cells because it might function in the nucleus. To answer this question, we obtained conditioned medium from htNSC expressing PTMS-HA versus Control-HA, and then added to the culture of various other type cells including hypothalamic neuronal line GT1-7, fibroblasts, hepatic cell line HepG2, and kidney cell line HEK293, as elucidated in Fig 2D. After 24 h of incubation, we used HA immunostaining to examine if HA tag could be present in these cells. The results showed that PTMS-HA was prominently transferred into all these cells, but Control-HA did not at all (Fig 2E). According to DAPI staining, HA-tagged PTMS entered and accumulated in the nuclei of these various cell types (Fig 2E). Because only some cells captured PTMS, it can suggest that a particular stage of cell cycling is needed for these cells to receive PTMS. Additional analysis showed that this nuclear transfer occurred very rapidly, as HA was detectable within 15 min in various recipient cells such as GT1-7 cells, HEK293 cells, and HepG2 cells (Fig 2F–H). Hence, agreeing with the biochemical information that PTMS contains a nuclear-localization signal (Kondili et al, 1996; Trompeter et al, 1996), our data suggest that this secretory protein can travel and cross the plasma membrane, traffic through the cytoplasm, and enter the nucleus of various recipient cells.

### PTMS transfer from htNSC to neurons in the hypothalamus

**Figure 3.**
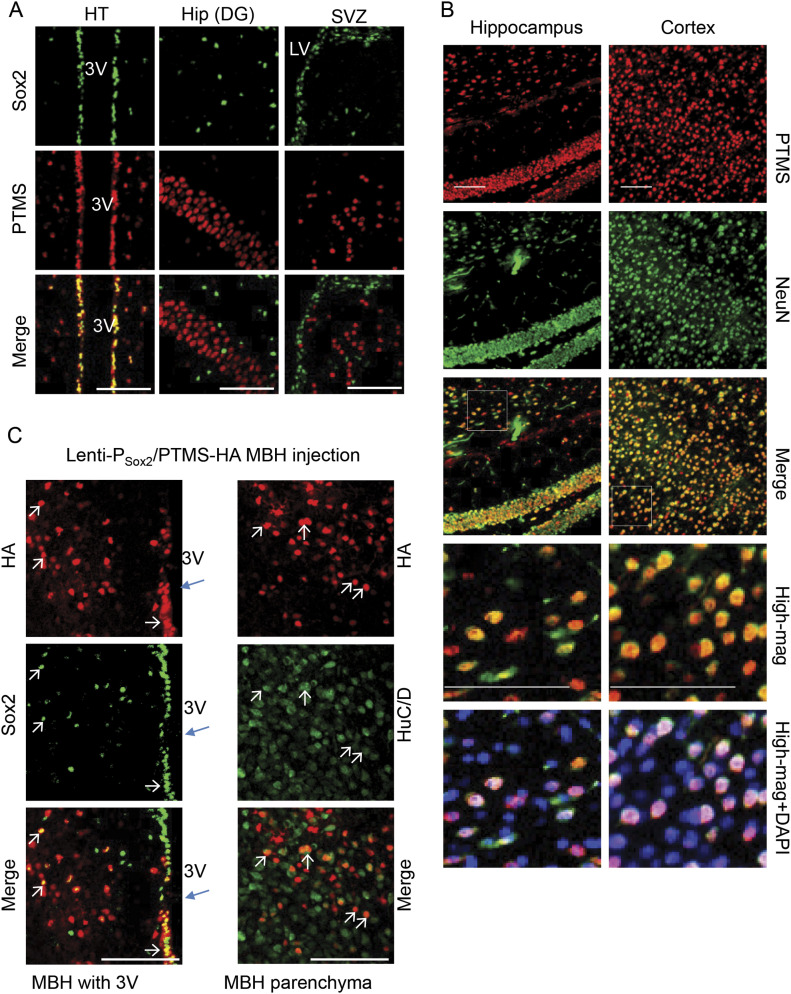
PTMS transferring from htNSC to neurons in the hypothalamus. **(A)** Representative brain sections of 3-mo C57BL/6 mice were immunostained for PTMS and NSC marker Sox2, focusing on NSC-positive regions including the third ventricle (3V) region of the hypothalamus (HT), dentate gyrus of hippocampus (Hip), and sub-ventricle zone of forebrain (SVZ). DAPI nuclear staining was included. **(B)** Brain sections across the cortex or the hippocampus of 3-mo male C57BL/6 mouse were generated and co-immunostained for PTMS and neuronal marker NeuN. Representative regions of parietal cortex and hippocampal dentate gyrus are presented. High-magnification views (High-mag) of outlined regions in merge images were included to show more details. **(C)** 3-mo C57BL/6 mice received unilateral injection of Sox2 promoter-driven PTMS-HA lentivirus in the mediobasal hypothalamus (MBH). Brain sections were made at day 7 postinjection and immunostained with Sox2 and neuronal marker HuC/D. 3V region of MBH containing Sox2-positive cells and MBH parenchyma containing HuC/D-positive neurons are presented in left and right panels, respectively. Small arrows pointed to co-localization of HA with Sox2 (left panel) or HuC/D (right panel). Scale bar, 100 μm.

**Figure S4.**
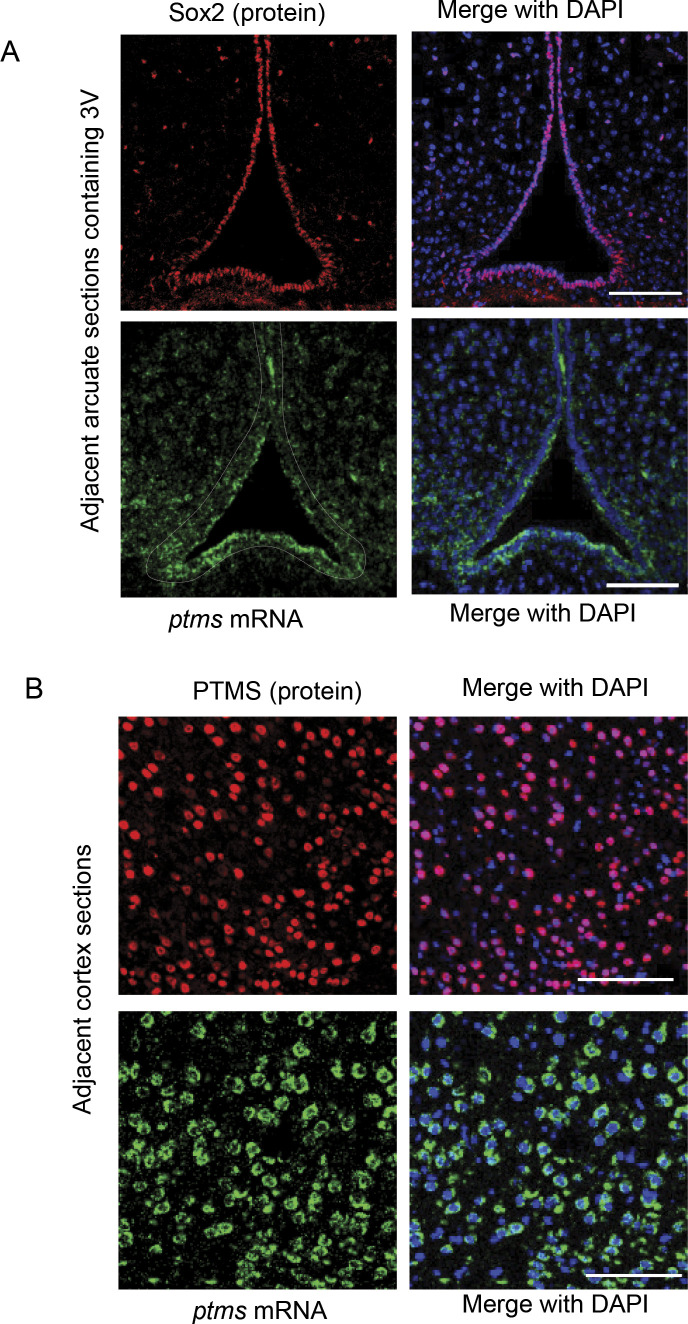
*ptms* mRNA expression in both htNSCs and cortex. **(A)** Hypothalamic sections of 3-mo male C57BL/6 mice were immunostained with NSC marker Sox2 and the adjacent sections were in situ hybridized with *ptms* mRNA probe (RNAscope). **(B)** Cortex sections of 3-mo male C57BL/6 mice were immunostained with anti-PTMS protein antibody or in situ hybridized with *ptms* mRNA probe. Scale bar, 100 μm.

As revealed previously, htNSC are Sox2 positive, mostly located in the hypothalamic third ventricle wall and the MBH parenchyma (Li et al, 2012, 2014; Zhang et al, 2017; Cai & Khor, 2019; Tang et al, 2020; Xiao et al, 2020). Consistently, the cerebroventricular system has been increasingly recognized for the role of neuropeptide in physiological regulation (Noble et al, 2018). From the results of Sox2 and PTMS co-immunostaining of mouse brain sections, PTMS was present in the nucleus of all htNSC (Fig 3A). We confirmed that this PTMS antibody was suitable for immunostaining, as it did not generate any specific signals when brain sections from *ptms* knockout mice were used. Because the hippocampal dentate gyrus and the subventricular zone in mouse brain are also known to contain adult NSCs, we examined these cells but found that PTMS was absent in either of them (Fig 3A). This difference can suggest that there are substantial differences between htNSC and other NSC types. On the other hand, in addition to htNSC, PTMS protein was found positive in majority of neurons throughout the brain and particularly in the cortex and hippocampus (Fig 3B). Using in situ hybridization, we also confirmed that *ptms* mRNA was detectable in htNSC as well as neurons in various brain regions (Fig S4). It is possible that PTMS is expressed in non-neuronal cells such as astrocytes and microglia (which was not addressed in this study because of technical limitation), but based on morphology, neurons are clearly important for abundantly expressing PTMS. Because of the feature that PTMS is transferable among cells, an interesting question was if htNSC could donate PTMS to neurons in vivo. To answer this question, we generated a lentivirus expressing PTMS-HA driven by Sox2 promoter, using a lentiviral promoter system which is effective for inducing gene expression specifically in Sox2-positive cells which belong to the populations of NSCs and early offspring before terminal differentiation. This method has been established in the literature and our previous studies (Li et al, 2012, 2014; Zhang et al, 2017; Tang et al, 2020; Xiao et al, 2020), as we repeatedly confirmed that this Sox2 promoter-driven lentivirus delivered an ectopic protein such as GFP specifically into Sox2-positive cells without targeting neurons. We delivered these lentiviruses into the mediobasal hypothalamus (MBH) of mice; at 1 wk after injection, hypothalamic sections were made for examining the expression of the delivered gene. Indeed, lentiviral induction of PTMS-HA led to HA signals in Sox2-positive cells (mostly in the 3V wall in the MBH, Fig 3C left). On the other hand, HA signals were also widely detected in many Sox2-negative cells (mostly in nuclei in MBH parenchyma, Fig 3C right); through neuronal HuC/D immunostaining, we confirmed that these cells were neurons. This observation was consistent with the in vitro data presented in Fig 2, suggesting that PTMS is not only secretory by htNSC but transferable from htNSC to neurons in the hypothalamus.

### Contribution of the hypothalamus to PTMS in the CSF

**Figure 4.**
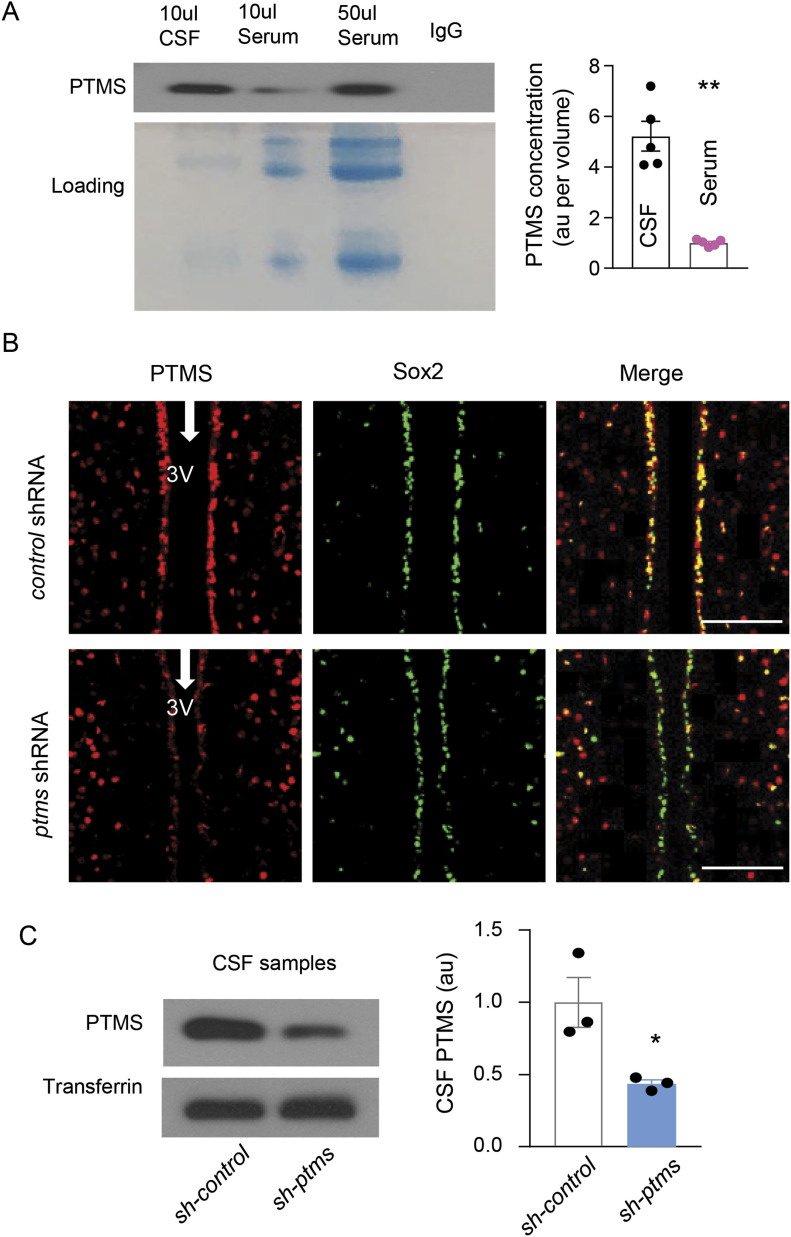
Contribution of the hypothalamus to PTMS in the CSF. **(A)** PTMS levels in blood serum versus CSF were detected by immunoprecipitation (IP)-Western blot (left panel), and the band intensities (au, arbitrary unit) were quantified (right panel). Matched IgG was used as a negative control of IP procedure, and Commassie staining was used as a loading control. **(B, C)** Injection of *ptms*-shRNA (*sh-ptms*) or scramble control shRNA (*sh-control*) lentiviruses into hypothalamic third ventricle of 3-mo male C57BL/6 mice (B), and at 1 mo postinjection, PTMS levels in CSF were measured by Western blot (left panel, transferrin as a loading control) and quantified (right panel). Scale bar, 100 μm. **(A, C)** *P < 0.05 (two-tailed unpaired *t* test), n = 4 mice (A) or 3 mice (C) per group; values represent the mean ± SEM.

**Figure 5.**
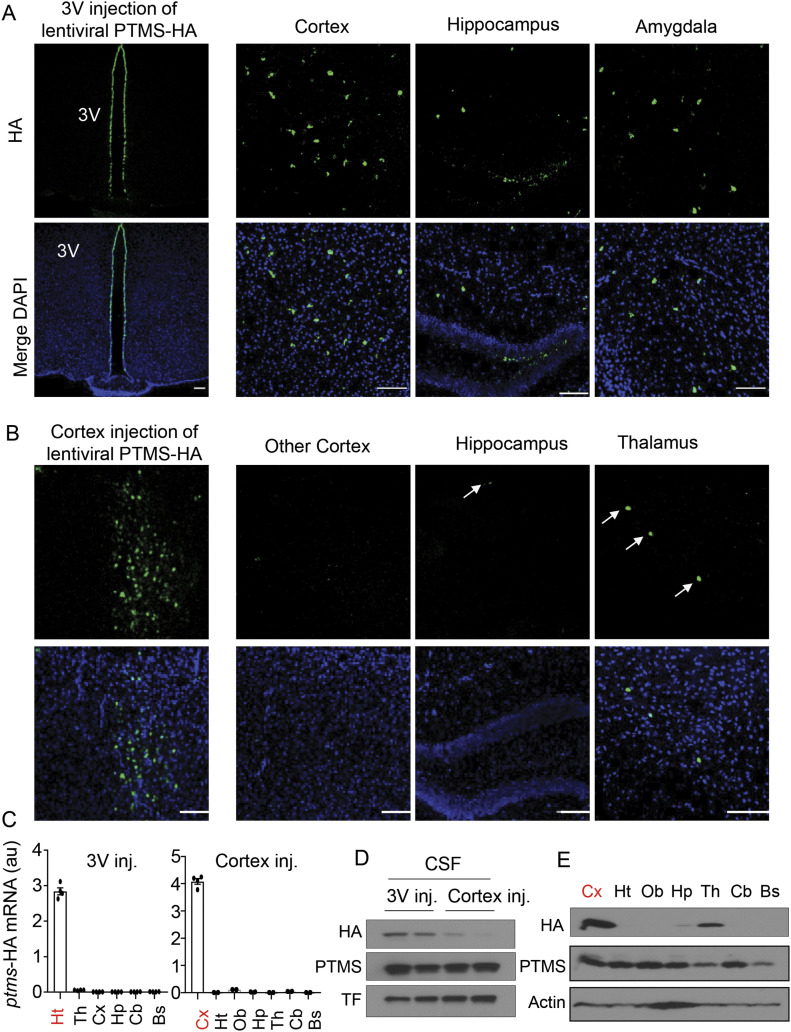
Transfer of hypothalamus and cortex PTMS to other brain regions. 4-wk male C57BL/6 mice received hypothalamic third ventricle or cortex injection of CMV-promoter driven PTMS-HA versus Control-HA, and at 2 wk postinjection, brain and cerebrospinal fluid (CSF) samples were collected for following analyses. **(A, B)** Induction of PTMS-HA in hypothalamic third ventricle wall (A) and parietal cortex (B) as revealed via HA immunostaining. Small arrows pointed to some HA positive cells in transfer brain regions. Scale bar, 100 μm. **(C)** Measurement of exogenous HA-tagged *ptms* mRNA by qRT-PCR for the injection brain region and other brain regions. **(D)** Measurement of PTMS in the CSF by HA and PTMS Western blots. Transferrin (TF) was used as a loading control. **(E)** Multiple brain areas of cortex injection group were examined for PTMS-HA expression across various brain areas. Scale bar, 100 μm. Ht, hypothalamus; Hp, hippocampus; Th, thalamus; Cx, cortex; Cb, cerebellum; Bs, brain stem. Additional information of control lentivirus injections are presented in Fig S5. n = 4 mice (C) per group; values represent the mean ± SEM.

**Figure S5.**
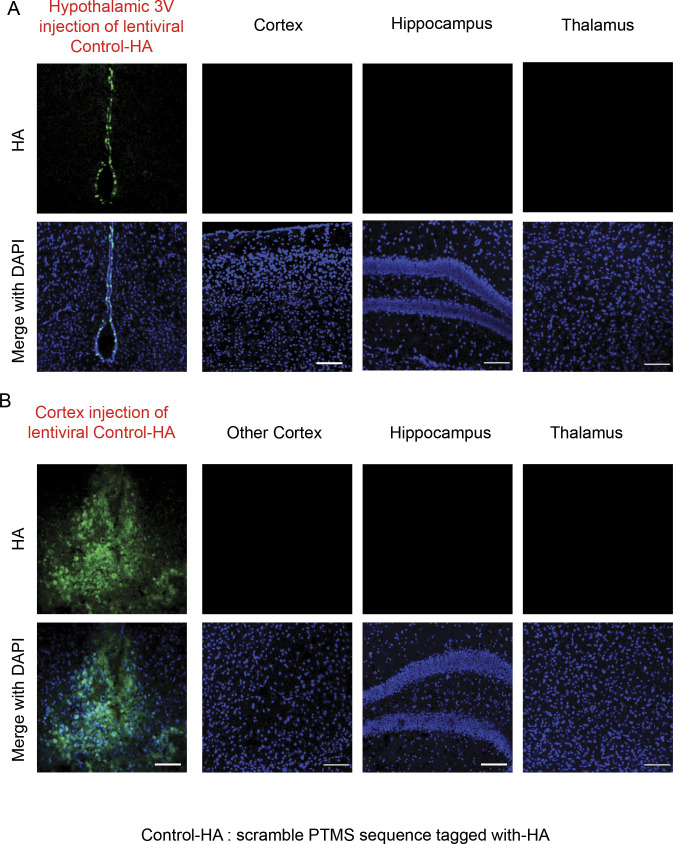
HA signals of the hypothalamic 3V and cortex control lentivirus injection. **(A, B)** 4-wk male C57BL/6 mice were injected with 0.4 μl CMV promoter-driven Control-HA lentivirus in hypothalamic third ventricle (A) or parietal cortex (B). At 2-wk post injection, brain sections of injected regions versus other brain areas were collected and analyzed via HA immunostaining. Scale bar, 100 μm.

We then measured PTMS levels in the cerebrospinal fluid (CSF) of mice, and its blood level was measured for a comparison. As shown in Fig 4A, PTMS concentration in the CSF is five times higher than that in the blood serum. In this context, we asked if hypothalamic PTMS release especially through htNSC could be important for its level in the CSF. In our previous research (Zhang et al, 2017), we have appreciated that lentiviruses when injected into the hypothalamic dorsal third ventricle infect only the cells on the surface but barely penetrated into the parenchyma or travel to other brain regions. To address this question, we performed an experiment by injecting lentiviruses of *ptms* shRNA versus scramble control into the hypothalamic third ventricle of mice, resulting in a substantial knockdown of PTMS expression in htNSC on the third ventricle surface compared with the control group (Fig 4B). Importantly, we found that this knockdown led to ∼40% drop of PTMS concentration in the cerebrospinal fluid (Fig 4C). Hence, although htNSC in the brain is rather a small population, it plays an important role for maintaining PTMS level in the cerebrospinal fluid. For comparison, a cortex injection of lentiviruses was performed in which PTMS-HA versus Control-HA were controlled by CMV promoter. Indeed, the hypothalamic third-ventricle injection led to HA expression specifically in Sox2-positive htNSC on the surface of third-ventricle wall in both groups, as represented by PTMS-HA group in Fig 5A and Control-HA group in Fig S5A. Injection of these PTMS-HA and Control-HA lentiviruses in the cortex is presented in Figs 5B and S5B, respectively. Using qRT-PCR primers which recognized only exogenous *ptms*-HA, we confirmed that *ptms*-HA mRNA was induced specifically in the hypothalamus or the cortex (Fig 5C). We obtained both CSF samples and brain sections from these mice for HA Western blot and immunostaining. As shown in Fig 5D, the hypothalamic injection of PTMS-HA led to an evident induction of this protein in the cerebrospinal fluid, but this effect was much weaker when cortex injection was performed. HA immunostaining showed that hypothalamic injection of PTMS-HA led to more appreciable numbers of HA-positive cells in other brain regions than did by cortex injection (Fig 5A and B), whereas Control-HA was absent in any of these brain regions (Fig S5A and B). Through Western blot, we were still able to detect a level of PTMS-HA in a few other brain regions such as the thalamus (Fig 5E), suggesting that PTMS from the cortex was transferable although to a limited extent. Taken together, PTMS from the brain and particularly the hypothalamus provides an important contrition to its level in the CSF.

### PTMS transfer among neurons in different brain regions

**Figure 6.**
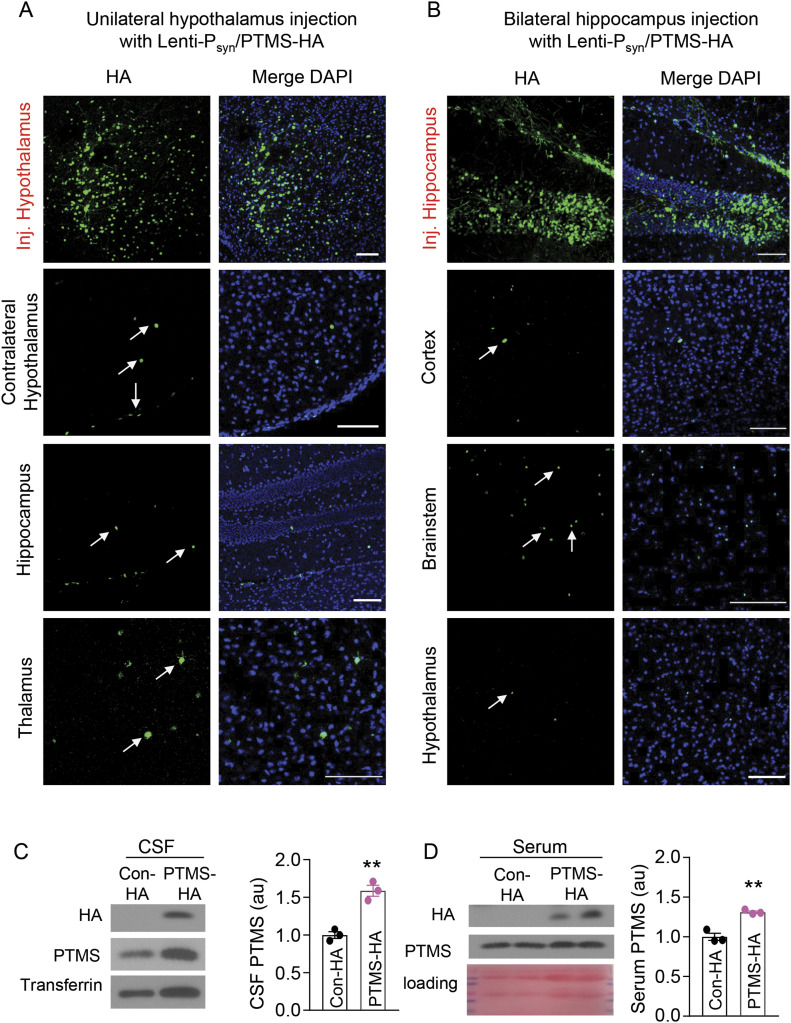
Transfer of hippocampal/hypothalamic PTMS to other brain regions and the blood. **(A, B)** 3-mo male C57BL/6 mice were injected with a dose of synapsin promoter-driven PTMS-HA lentivirus in one side of the posterior hypothalamus (A) or both sides of the hippocampus targeting CA1 and dentate gyrus (B). At 2 wk post injection, brain sections were immunostained for HA in the injected regions versus other brain areas. Small arrows pointed to some HA positive cells in transfer brain regions. Scale bar, 100 μm. Additional information of control lentivirus injections are presented in Fig S6. **(C, D)** 3-mo male C57BL/6 mice received bilateral injections of neuron-specific lentivirus of synapsin promoter-driven PTMS-HA (labelled as “PTMS-HA”) versus Control-HA (labelled as “Con-HA”) in the hippocampus (targeting CA1 and dentate gyrus) and posterior hypothalamus, and at 2-wk post injection, secretion of PTMS-HA to the CSF (C) and the serum (D) was assessed via IP-Western blotting. **(C, D)** Transferrin blot (C) or Ponceau S staining (D) was used as loading control. **P < 0.01 (two-tailed unpaired *t* test), n = 3 mice per group; values represent the mean ± SEM.

**Figure S6.**
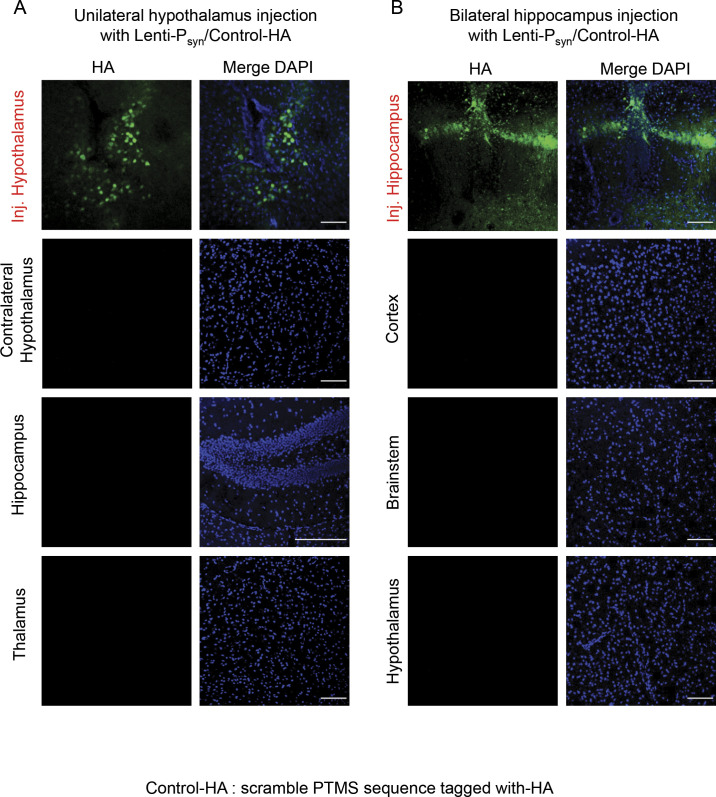
HA signals of the hypothalamus and hippocampus control lentivirus injection. **(A, B)** 3-mo male C57BL/6 mice were injected with a dose of synapsin promoter-driven Control-HA lentivirus in one side of the posterior hypothalamus (A) or both sides of the hippocampus targeting CA1 and dentate gyrus (B). At 2 wk post injection, brain sections were immunostained for HA in the injected regions versus other brain areas. Scale bar, 100 μm.

Because we observed that neurons also produce PTMS, a relevant question was, if neurons could act as not only recipients but also donors of PTMS? We have obtained some initial information from cortex injection in Fig 6, but because these lentiviruses were not neuron specific, we decided to use a neuron-specific lentivirus for independent experiments. Therefore, PTMS-HA and Control-HA lentiviruses were made under neuronal synapsin promoter and were injected into the hypothalamus versus hippocampus, as represented in Figs 6 and S6. We found that hypothalamic and hippocampal neurons were both able to transfer PTMS into a few different brain regions (Fig 6A and B). PTMS transfer from hypothalamic neurons was generally stronger than that from hippocampal neurons, agreeing with the classical endocrine role of the hypothalamus. In summary, consistent with being a special type of hypothalamic endocrine cells, htNSC are important for endocrine release of PTMS, whereas brain neurons seem to have a secondary contribution to this process.

### PTMS transfer from the brain to the blood

Considering this gradient between the CSF and blood, we asked if brain PTMS could provide a contribution to blood PTMS. To answer this question, we used lentiviral injection to overexpress PTMS-HA in both the hippocampus and hypothalamus of mice and then examined if this HA-tagged peptide could be detected in the blood of these animals. Using qRT-PCR primers which specifically detected exogenous tagged *ptms*, we confirmed that injection of lentiviral PTMS-HA increased the mRNA levels of exogenous HA-tagged *ptms* only in the brain without any leakage into the peripheral tissues. HA-tagged PTMS was readily detectable in the CSF through HA immunoblotting (Fig 6C). This resulted in ∼60% increase in PTMS level in the CSF (Fig 6C). Thus, agreeing with the fact that the size of neuronal population is much larger than that of htNSC, the total contribution of neuronal PTMS to the CSF is substantial. Excitingly, we observed that HA-tagged PTMS was present in the blood of these mice (Fig 6D), which led to ∼25% increase in blood PTMS level compared with the control group (Fig 6D). Hence, because PTMS has been known to be produced and released by peripheral organs such as the liver, our data suggest that although the peripheral production of PTMS probably contributes to its baseline level in the blood, the brain can sensitively modulate the blood level of PTMS. This finding suggests there is a top-down regulation from the brain to the blood through neuronal release of PTMS. Altogether, apart from the hypothalamus known as the endocrine headquarters, our finding suggests that the brain is an endocrine organ through secreting a protein to regulate the brain itself as well as the periphery.

### Declines in brain and hypothalamic PTMS during aging

**Figure 7.**
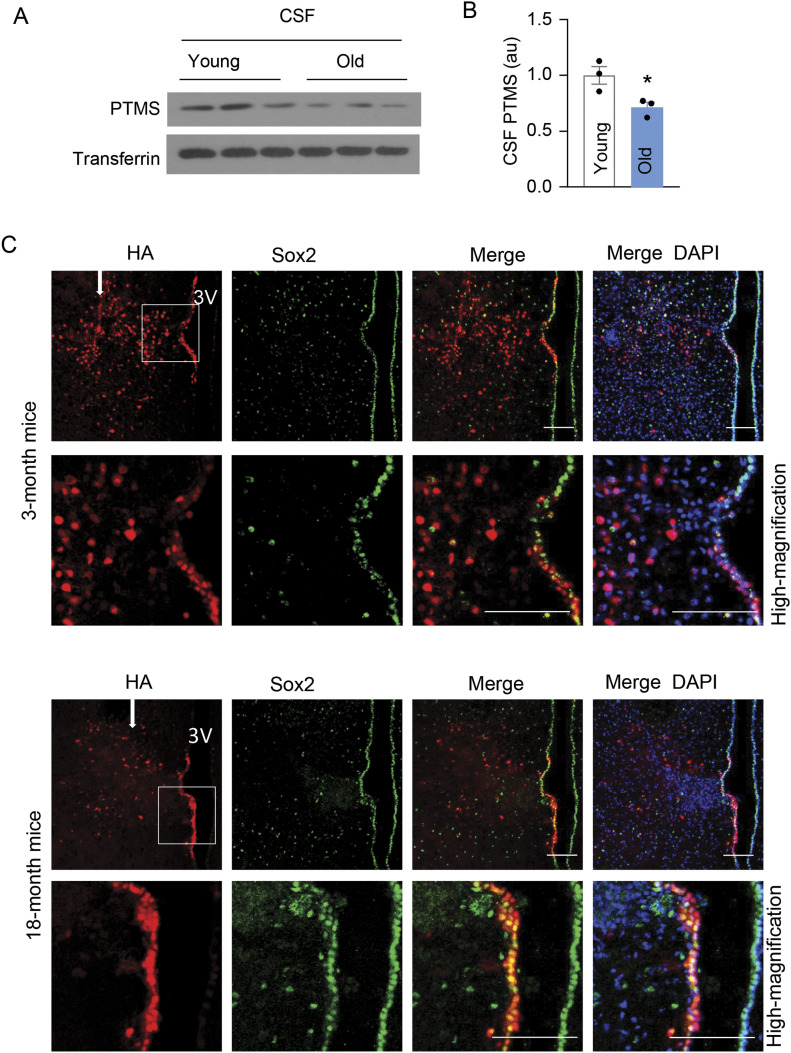
PTMS secretion declines during aging. **(A, B)** PTMS levels in the CSF of male C57BL/6 mice at young (∼4 mo) or old (∼24 mo) age were measured by Western blot (A) which were quantified in panel (B). Transferrin was used to as a loading control. **(C)** Young (3-mo) or middle aged (18-mo) male C57BL/6 mice received unilateral injection of the same dose of Sox2 promoter-driven PTMS-HA lentivirus in the mediobasal hypothalamus. White arrows point to the site of injections. At 1 wk postinjection, hypothalamic sections were generated for HA immunostaining to show the levels of both primary expression of PTMS-HA in Sox2-positive cells and secondary transfer of PTMS-HA into Sox2-negative cells. Sox2 co-immunostaining was performed to reveal Sox2-positive cells in this region. Lower panels in show high-magnification views of outlined areas. Scale bar, 100 μm. 3V: hypothalamic third ventricle. *P < 0.05, two-tailed unpaired *t* test (B), n = 3 mice per group; values represent the mean ± SEM.

Based on in vitro finding that PTMS works to protect against cell-replicative aging (Fig 1), we were interested in investigating if this protein could be significant for physiological aging. To provide another rationale, we measured PTMS levels in the CSF of young versus old mice. As shown in Fig 7A and B, PTMS levels in the CSF of old mice declined compared with the levels in young mice. These declines could be partially attributed to the fact that htNSC are significantly lost during aging as we have appreciated previously (Zhang et al, 2017). Apart from the effect due to htNSC loss, we also examined if dysfunctional htNSC in aging represents another reason for PTMS decline in the brain. In this experiment, we compared young versus middle-aged mice with lentiviral overexpression of HA-tagged PTMS in Sox2-positive htNSC in the mediobasal hypothalamus. We performed a single injection of these PTMS-HA lentiviruses into the mediobasal hypothalamus. At 1 wk after microinjection, we analyzed HA signals in the brain sections of these mice. In agreeing with aging-associated loss of htNSC, lentiviral induction of HA signals was indeed much less abundant in the mediobasal hypothalamus and its third ventricle wall of old mice compared with young mice (Fig 7C). Notably, when HA-tagged PTMS was abundantly transferred to neurons in young mice, this activity became almost absent in old mice (Fig 7C). Thus, compared with young htNSC, aged htNSC are dysfunctional in producing, secreting, or transferring PTMS. Hence, physiological aging is associated with a dramatic decrease in PTMS secretion by htNSC.

### PTMS loss in the hypothalamus versus hippocampus in affecting aging behavior

**Figure 8.**
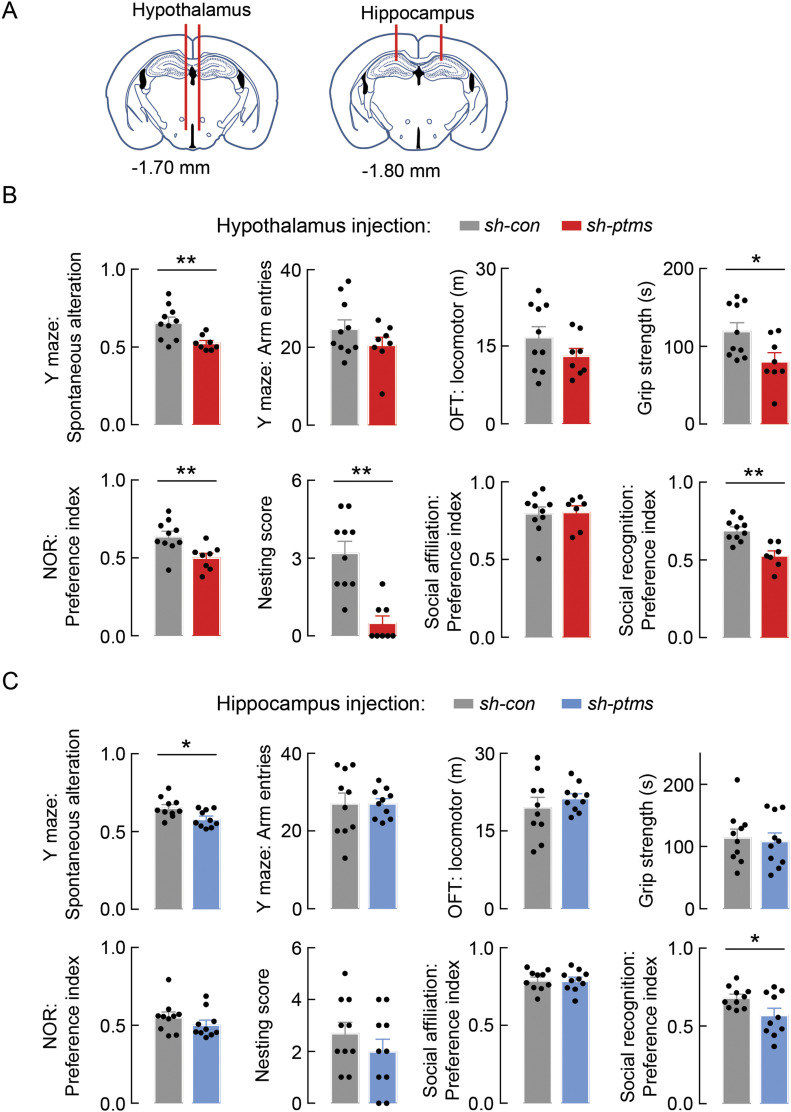
PTMS loss in the hypothalamus or hippocampus leads to aging-related behaviors. Middle-aged male C57BL/6 mice (15 mo old) were bilaterally injected with lentiviruses of scramble control shRNA (labelled as “*sh-con*”) or *ptms* shRNA (labelled as “*sh-ptms*”) in the hypothalamus, hippocampus. **(A)** Injections targeted posterior hypothalamus, CA1 and dentate gyrus of hippocampus (bregma distance in each injection is labelled), as elucidated in (A). **(B, C)** Behavioral tests were performed at 5 ∼ 6 mo post viral injection (B, hypothalamus injection; C, hippocampus injection). *P < 0.05, **P < 0.01, two-tailed unpaired *t* test, n = 7–10 mice per group; values represent the mean ± SEM.

Because the brain is responsible not only for PTMS level in the CSF but also has a contribution to the blood PTMS level, we further evaluated the prediction that the brain or certain brain regions should have a critical role for anti-aging effects of PTMS. To do so, we first employed several knockdown mouse models via bilateral micro-injection of *ptms* shRNA lentiviruses into different brain regions of middle-aged male C57BL/6 mice. As elucidated in Fig 8A, same dose of *ptms* shRNA versus control scramble shRNA lentiviruses were bilaterally injected to each of these brain regions using the same cohort of age- and conditions-matched mice. After injections, these mice were longitudinally monitored for aging-related behavioral dysfunctions through a batter of noninvasive behavioral assays, including Y-Maze, grip test, open field test, novel object exploration, cognition, sociality, and nest building tests. No evident differences were found in either knockdown group compared with their matched controls at 2 mo after injection. However, at 5 ∼ 6 mo after injection, we found that hypothalamic *ptms* knockdown mice performed much worse than the controls across almost all these behavioral tests (Fig 8B). Hippocampal *ptms* knockdown mice performed worse in a few hippocampus-dependent behaviors including Y-maze and social recognition but did not affect other behavioral functions compared with the controls (Fig 8C). Although there could be many additional experiments to compare other aspects of aging, this behavioral difference represented a piece of valuable information to suggest that loss of PTMS in the brain provides a contribution to aging physiology, and such a functional loss in the hypothalamus is relatively more important which agrees with the role of hypothalamus in endocrine control of aging.

## Discussion

Recently, the hypothalamus was found to play a role in health and lifespan control (Satoh et al, 2013; Zhang et al, 2013), and htNSC was shown to be responsible at least through the secretion of miRNA-containing exosomes (Zhang et al, 2017). This previous knowledge suggested that htNSC have special endocrine functions in influencing aging physiology, but the endocrine secretion portfolio of these cells remained to be mapped. In this study, we performed protein screening using an in vitro co-culture model and found that htNSC secrete PTMS to prevent against cell senescence of fibroblasts in the co-culture system. Biochemically, PTMS is conserved across species and could work in the nucleus according to its sequence and some biochemical assay (Kondili et al, 1996; Trompeter et al, 1996); however, the biological significance of this protein is barely known. Here, we discovered that PTMS is evidently released by htNSC and is quickly transferred into the nuclei of other cells and particularly neurons in various brain regions. These findings further support that htNSC have special endocrine functions more than neurogenesis as its name suggested, a point which we began to elucidate in our previous studies (Zhang et al, 2017; Cai & Khor, 2019; Tang et al, 2020).

Interestingly, neurons in various brain regions not only receive PTMS but also release PTMS and thus contribute to its levels in the CSF. Compared with various brain regions, the hypothalamus is relatively stronger in inducing PTMS transfer over a long distance, for example, from the hypothalamus to the cortex. This advantage of the hypothalamus is likely attributed to htNSC which do not exist in other brain regions. On the other hand, because the size of neuronal populations throughout the brain is much larger than that of htNSC, the total release of PTMS from neurons is immense, which can lead to a contribution to the blood level of PTMS. Thus, our study indicates that neuroendocrinology should not limited to a few small brain regions such as the hypothalamus but can be expanded to the majority (if not all) of neurons in the brain although they have been classically known for inducing synaptic transmission. However, the brain and the hypothalamus within the brain should represent different orders in performing endocrine functions. We propose a conceptual model that whereas the hypothalamus is a primary-order neuroendocrine organ, the rest of the brain is a second-order neuroendocrine organ which is influenced by the hypothalamus but can independently perform an endocrine function.

It is still unclear how PTMS works in the nuclei of different cells, but likely it might provide an effect through stabilizing some nuclear and chromatin structures, given that it was previously shown to bind to histone (Kondili et al, 1996). It also remains to be determined if a carrier should be needed for PTMS release and transfer, especially considering that PTMS is an acidic protein which logically might not be circulated and transferred freely by itself. We have obtained some results showing that extracellular vesicles (exosomes and microvesicles) of htNSC contain PTMS; however, to study this possibility is a different subject, and besides, we also recognize that extracellular vesicles might not be the only way involved.

Physiologically, we generated mouse models with *ptms* knockdown specifically in the hypothalamus versus hippocampus and the results indicated that PTMS is neuroprotective while the hypothalamus played a more overall role in supporting neurobehavioral functions. These studies, albeit limitedly, provided initial proof-of-concept evidence to suggest that PTMS secretion and transfer are physiologically important, whereas much more studies are needed to demonstrate the details. We also predict that PTMS secretion and transfer are important not only for the brain but also for peripheral tissues, but it remains to be studied if brain versus peripheral secretion of PTMS might cooperatively work to regulate whole-body physiology, and if the hypothalamus-brain orchestration might provide a regulatory role in this process.

## Materials and Methods

### Cell culture

Primary culture of htNSC was performed as described previously (Li et al, 2012; Zhang et al, 2017; Tang et al, 2020). In brief, the hypothalamus was dissected from newborn C57BL/6 mice, cut into small pieces of ∼1 mm^3^, and followed by digestion using TrypLE Express enzyme (Life Technologies) for 10 min at 37°C. After centrifugation, cells were suspended in NSC medium composed of neurobasal-A (Life Technologies), 0.25% GlutaMAX supplement (Life Technologies), 2% B27 without vitamin A (Life Technologies), 10 ng ml^−1^ EGF (Sigma-Aldrich), 10 ng ml^−1^ bFGF (Life Technologies), and 1% penicillin–streptomycin and seeded in ultralow-adhesion six-well plates (Corning). 1 wk later, neurospheres were collected by centrifugation and trypsinized with TrypLE Express enzyme into single cells, passaged, and maintained in neurosphere culture until experimental use. MSFs were isolated from mice at postnatal day 7–14 with the enzymatic method as described (Rittié & Fisher, 2005). Briefly, the mice were euthanized, and shaved, washed, and sterilized with 70% EtOH and thoroughly rinsed with PBS. A longitudinal dorsal incision was made on the skin from the tail to the snout, followed by incision around the truncus and whole skin removal. Several skins were placed with dermis-side down and floated onto 0.25% trypsin solution for overnight incubation at 4°C. The dermis was peeled off from the epidermis with forceps, minced into small pieces (<1 mm) if necessary, and collected into DMEM medium with 10% FBS (growth medium). The dermis was then vigorously vortexed for 20–30 s and filtrated through a 10-μM nylon cell strainer (Falcon), and the recovered cells from the filtrate were plated onto cell culture dishes in growth medium and passaged every 3 d with 0.25% trypsin. HEK293T cells (CRL-3216), HEK293 (CRL-1673), and HepG2 (HB-8065) were purchased from ATCC, and GT1-7 cells were established as previously reported (Zhang et al, 2013), those cells were cultured in DMEM containing 10% fetal bovine serum and 1% penicillin–streptomycin. All cell lines used in this study were tested free of microbial (including mycoplasma) contamination and their morphology and growth characteristics were compared with published information to ensure their authenticity. Conditioned medium for the proteomic analysis and for the fibroblast treatment were generated in the same way.

#### htNSC and fibroblast co-culture

On the day before co-culturing, htNSC were passaged in ultra-low attachment dishes to form neurospheres in neural basal A medium plus B27 without antioxidants, and 10^4^ MSFs were seeded on the bottom of 12-well transwell (#3460; Corning). On the day of co-culturing, neurospheres or blank medium control were transferred to the insert of transwell (upper compartment) and were replaced with fresh neurospheres or blank medium control every 2 or 3 d. Neurospheres always contained the indicated numbers of htNSC based on cell counting of aliquots. MSF at the bottom were trypsinized and counted when the confluency reached 80–90%, and 10^4^ cells were replated. MSF were exposed to the same co-culturing conditions at each passage. These MSF grew similarly in the neurophere medium compared with standard fibroblast medium. The population doubling number of MSFs was calculated using the forsmula ΔPDL = log(n_f_/n_0_)/log_2_, where N_0_ is the initial number of cells that were seeded and n_f_ is the final number of cells that were harvested.

#### Stable shRNA-expressing htNSC lines

Using the method as previously described (Li et al, 2012; Zhang et al, 2017), various shRNA lentiviruses-infected htNSC were selected with puromycin at 1 μg/ml for 7–10 d, and then maintained in regular culture medium supplemented with 0.5 μg/ml puromycin for maintenance.

### Plasmids and recombinant lentiviruses

Various versions of lentiviral shRNA vectors and controls were purchased from Sigma-Aldrich, including PTMS shRNA (TRCN0000346743), PPIA/CyPA shRNA (TRCN0000101190), NDKA shRNA (TRCN0000360520), Cystatin C shRNA (TRCN0000055249), Pleiotrophin shRNA (TRCN0000071676), Hist1h2bb shRNA (TRCN0000093066), IGFBP-2 shRNA (TRCN0000012859), and Ube2V1 shRNA (TRCN0000039509). The plasmid plenti-CMV-*ptms*-HA was obtained by inserting coding sequence of *ptms*-HA into the plenti6 backbone (Invitrogen) with *BamHI* and *XhoI* sites. HA was tagged at the C-terminal of PTMS to protect its N-terminal which involves in mediating its secretion. Size-matched control sequence was based on scrambled version of *ptms* which was synthesized and verified by Genewiz, and was inserted into the plenti6 backbone to generate plenti-CMV-control-HA. The scrambled control sequence of *ptms*: 5-GGATCCGCCACCATGCGCGAAGAAAAAGAAGATGAAAAAGATGAAGATGCGATGCTGAAACCGGAAGATAACGGCGAAGAAAAAGAAGCGGATGAAGTGGAAGGCGATACCGCGAAAGAAGATGAAACCCTGGCGGAAGCGGAACGCAGCGAAAAAGAAAAAGAAGAAGGCGCGGCGGAAGAAGCGGATGAACGCGAAAAAGAAAAAGAAGAAAGCGAACGCACCGTGGATGAAGAAAACGAAAAAGAACAGAAAGGCAGCCCGGAAAGCAAAGCGAAAGAAGCGGGCGATGCGCGCGTGGTGGGCGTGGAAGAAGGAAGCGGATACCCATACGATGTTCCAGATTACGCTTGACTCGAG-3. The plasmid pSox2-*ptms*-HA and the scrambled control were obtained by replacing the Cre with *ptms* or its scrambled control sequence in the lentiviral plasmid pSox2-Cre which was described previously (Li et al, 2012; Zhang et al, 2017). Synapsin promoter-directed lentiviral vector were obtained by inserting *ptms* or its scrambled control sequence into the Synapsin lentiviral plasmid with *BamHI* and *PciI* sites. Lentiviruses were produced by transfecting HEK293T cells with corresponding viral and packaging plasmids, purified by ultracentrifugation and titrated using the p24 ELISA kit as described previously (Zhang et al, 2017).

### Cell senescence, size and secretome assays

#### Cell senescence analysis

Senescence in cell culture was determined by Senescence β-Galactosidase (SA-β-Gal) Staining Kit (#9860; Cell Signaling) according to the manufacturer’s instruction as previously described (Tang et al, 2013). The developed blue color on cells was captured with the Zeiss Axiocam HRc camera of Zeiss Observer at the imaging core facility at the Albert Einstein College of Medicine.

#### Cell size profiling

Cells were trypsinized into single cells and mounted on a hemocytometer, and cell images were captured with a standard light microscope. The pixel area of each cell on the image was calculated with ImageJ. Briefly, 8-bit images were thresholded with B&W default setting and processed with binary functions “Fill Holes” and “Watershed” in sequence. The pixel area of each cell-shaped particle was calculated with “Analyze Particles” function. Only particles within a size range of “80-Infinity” were included to avoid the interference of cell debris. To plot the cell size frequency distribution, pixel areas of at least 100 cells from 4 to 5 random fields (randomly chosen microscopic fields containing at least 20 cells per field) were used to calculate the bin and frequency of the histogram with Data Analysis ToolPak in Excel.

#### Cell secretome analysis

5 × 10^5^ htNSC were seeded in six-well ultra-low attachment plate and cultured for 24 h. 30 μl of Blank or conditioned medium was subjected to SDS–PAGE, and the polyacrylamide gel was stained by silver staining kit (Sigma-Aldrich) according to the manufacturer’s instruction. A unique protein band of htNSC condition medium at about 15 kD in size was visually identified, and excised and analyzed by nanoLC-MS/MS using a Waters nanoAcquity and Orbitrap Velos mass spectrometer (Thermo Fisher Scientific) at the proteomic facility of Albert Einstein College of Medicine. Protein identification was performed using Mascot with precursor and product ion tolerances of 25 ppm and 0.4 kD, respectively; carbamidometyl Cys as a fixed modification and deamidation (Asn & Gln), and oxidation (Met) as variable modifications against the SwissProt database.

### PTMS antibody generation

Customized PTMS antibody was collaboratively developed with Pierce Biotechnology, Inc. In brief, several different versions of 26-mer short peptides according to the predicted essential regions of PTMS were commercially synthesized and emulsified with Freund’s adjuvant to immunize rabbits for generating polyclonal anti-PTMS sera. All anti-PTMS sera were screened together using several methods including Western blots and ELISA as well as immunostaining for negative controls including various *ptms* knockout mouse samples and positive controls including HA-tagged PTMS expression in cells and tissues. A specific antibody was identified which was generated against the 26-mer peptide sequence of VEAAAELSAKDLKEKKDKVEEKAGRK which corresponded to the amino acids 6–31 of mouse PTMS. This anti-PTMS serum was subjected to IgG purification and further confirmed after purification for the specificity and quality before being used in this study.

### PTMS secretion and transfer assays in vitro

#### 

#### 

htNSC were transduced with CMV-*ptms*-HA versus matched control lentivirus for 24 h, and selected with blasticidin at 1 μg/ml for 7–10 d. The post-selected cells (donor) were then cultured in regular medium for 24 h, and the conditioned medium was added to the acceptor cell culture for various amount of time. The acceptor cells were examined by PTMS or HA immunofluorescence or Western blot to assess the intercellular PTMS transfer efficiency.

### Immunostaining

#### Cell immunostaining

Cells were seeded on laminin or polylysine-coated coverslips and fixed with 4% PFA for 15 min at room temperature before immunostaining. For cultured htNSC spheres, they were fixed with 4% PFA for 30 min at room temperature and equilibrated with 20–30% sucrose, and cryosections were made at a thickness of 10 μm.

#### Tissue immunostaining

Mice under anesthesia were transcardially perfused with 4% PFA, and then the brains and peripheral tissues were removed, PFA-fixed, and equilibrated with 20–30% sucrose, followed by cryosectioning at a thickness of 10 (brain) or 20 μm (peripheral tissues). Cell or tissue specimen were blocked with the serum of the appropriate species, treated with primary antibodies, including rabbit anti-PTMS, rabbit anti-HA (3724; Cell Signaling), mouse anti-Sox2 (MAB2018; R&D Systems), rabbit anti-Nestin (MAB353; Millipore), mouse anti-NeuN (MAB377; Millipore), and mouse anti-HuC/D (A21271; Invitrogen), subsequently incubated with Alexa Fluor 488 or 555–conjugated secondary antibodies. Technical controls for antibody reaction included using naive IgGs of the appropriate species. DAPI staining of sections was used to reveal all cells. Images were captured using Leica SP8 confocal microscope.

### Western blot and immunoprecipitation

Tissue protein lysate was prepared by sonication in ice-cold radioimmunoprecipitation assay (RIPA) lysis buffer (20 mM Tris-HCI, pH 7.4, 10 mM NaCl, 1 mM EDTA, 0.01% SDS, 1% Triton X-100, and 1× protease inhibitor cocktail). CSF was collected from anaesthetized mice by penetrating a pulled capillary tube into the cisterna magna through dura mater, as previous described (Zhang et al, 2017). Serum was prepared by collecting supernatant from fully clotted whole blood (1 h undisturbed at room temperature). CSF and whole blood was mixed with protease inhibitor cocktail immediately upon collection. The boiled protein samples were separated by 18% SDS–PAGE and were transferred onto 0.2 μm polyvinylidene difluoride membrane (PVDF) membrane in acidic buffer (20 mM sodium acetate buffer, pH 5.0) at 15V for overnight. The membranes were then fixed with 0.5% PFA in PBS for 15 min, washed with 50 mM glycine in PBS for 5 min, and blocked with 5% non-fat milk in TBST before immunostaining. Primary antibodies were rabbit anti-PTMS (1:2,000), rabbit anti-β-Actin (1:1,000, #4967; Cell Signaling), rabbit anti-HA (1:2,000, #3724; Cell Signaling) and rabbit anti-Transferrin (#NBP1-97472; Novus Biologicals) primary antibodies overnight at 4°C, followed by HRP-conjugated goat anti-rabbit secondary antibody (1:10,000; Cell Signaling). For immunoprecipitation, anti-PTMS antibody–coupled Sepharose beads were prepared as described in literature (Michielsen et al, 2020). Briefly, 100 mg protein A-magnetic Sepharose beads (#28951378; GE Healthcare) were washed with PBS and blocked with 1 g/l BSA (Sigma-Aldrich) for 1 h at room temperature, followed by incubation with 70 μg PTMS antibody dissolved in blocking buffer for 1 h at room temperature with rotation. After three washes with excessive PBS, the beads were cross-linked in 20 mmol/l dimethyl pimelimidate (Sigma-Aldrich) and 200 mmol/l triethanolamine (Alfa Aesar) in PBS solution (pH 8.6) for 30 min and washed with 200 mmol/l triethanolamine (Alfa Aesar) in PBS solution. After two additional repeats of cross-linking and washing, the beads were incubated in 50 mmol/l ethanolamine (Alfa Aesar) in PBS for 1 h at room temperature, and two washes with 1 mol/l glycine-HCl (pH 3.0) at 56°C, 20 min each. PTMS immunoprecipitation: serum, CSF or cell culture supernatant were mixed with anti-PTMS antibody coupled beads (1:25 for serum and CSF, 1:250 for cell culture supernatant, dry weight), and were rotated in PBST containing 1× proteinase inhibitor cocktail and 7 g/l nonfat milk for overnight at 4°C. Eluted proteins were SDS–PAGE separated and detected by Western blot as previously described. Ponceau S staining (#P7170; Sigma-Aldrich) of membrane or Coomassie G-250 staining (#161–0786; Bio-Rad) (#AG25; Sigma-Aldrich) of a duplicate gel was used as loading control.

### RNA assays

#### RNA in situ hybridization

A 16ZZ probe named Mm-*ptms* targeting 2–1,059 of NM_026988.2 was designed by Advanced Cell Diagnostics, and in situ detection of *ptms* mRNA transcription of fixed cyrostat sections using the RNAscope kit (Advanced Cell Diagnostics) was performed according to the manufacturer’s protocol. Positive control probe targeting housekeeping gene *Pplb* and negative control probe targeting bacterial DapB gene were included in each experiment, Images were captured using Leica SP8 confocal microscope.

#### RNA extraction and qRT-PCR

Total RNA from cells or tissue was extracted using TRIzol (Invitrogen) following the manual. cDNA was synthesized using the Moloney Leukemia Virus Reverse Transcriptase system (Promega). Real-time PCR was performed using the SYBR Green PCR Master Mix (Applied Biosystems). Relative gene expression levels were normalized against the mRNA levels of the house-keeping gene *Actb*.

### Mouse models

C57BL/6J mice were obtained from Jackson Laboratories and kept in standard, infection-free housing conditions, with 12 light/12 dark cycles and 4–5 mice per cage. The *Ptms* knockout mouse line was generated by Einstein’s transgenic core using CRISPR technology. All procedures were approved by the Institutional Animal Care and Use Committee of the Albert Einstein College of Medicine (protocol #20171210, #20171209, and #20171208).

#### Lentivirus injection

The following coordinates were used to target the hypothalamus (AP −1.7, ML ±0.25, DV −5.8), the hypothalamic third ventricle (AP −1.7, ML 0, DV −5.0), the hippocampus (AP −1.8, ML ±1.3, DV −1.5), and the parietal cortex (AP −1.8, ML ±2.0, DV −0.2). Lentiviruses in the dose of 200 ng p24/μl with artificial CSF (aCSF; Tocris Bioscience) were injected into brain regions with an ultra-precise stereotactic apparatus (David Kopf Instruments) via a 26-gauge guide cannula and a 33-gauge internal injector (Plastics One) connected to a 5-μl Hamilton syringe and infusion pump (WPI Instruments) (Zhang et al, 2017), all brain parenchyma viral injection volumes were 1-μl per injection site at rate, and the third ventricle injection and its comparative cortex injection volume was 0.4 μl per mouse.

### Behavioral tests

All behavior tests were performed in the behavioral testing room. An Anymaze video-tracking system (Stoelting) equipped with a digital camera connected to a computer was used for the whole course of animal activities in training and experimental sessions of behavioral tests as we described previously (Zhang et al, 2013), with minor modifications, including the following tests. (1) The grip test (muscle endurance): as described, a mouse was lifted by the tail and placed on a homemade square grid (1 cm mesh size), the grid was then inverted 30 cm over a soft pad, and the mouse was allowed to hang by paws for 5 min. Three repeats were performed for each mouse with at least 10 min rest between each trial. (2) Open field test: a mouse was placed into the lower right corner of a white plastic chamber (40 cm length × 40 width and × 40 cm height) facing to wall. The mouse was allowed to freely explore the chamber, and locomotor activity was monitored for 10 min. (3) Novel object recognition (Leger et al, 2013): a mouse was allowed to freely explore an open-field box for 10 min before experimental sessions. During the first session (familiarization session), the mouse was allowed to freely explore two similar objects, and during the second session (test session), one of the two objects was replaced by a novel object for 10 min. An intersession interval of 6-h between the two sessions were applied. The amount of time that the mouse spent on exploring each object was recorded. A preference index was calculated using the ratio of the amount of time spent exploring any one of the two objects (including novel one) over the total time spent exploring both objects. (4) Y maze spontaneous alternation (Miedel et al, 2017): testing occurred in a Y-shaped maze with three plastic arms at a 120° angle from each other. A mouse was placed into one arm facing the center junction zone, and allowed to freely explore the maze for 10 min. An arm entry occurred when all four paws of the mouse cross the arm-center zone boundary with the mouse’s snout oriented towards the end of the arm. A spontaneous alteration occurred when the mouse made three consecutive arm entries of the Y maze clockwise or counterclockwise. Spontaneous alternation = number of spontaneous alternations/(total number of arm entries − 2). (5) Social affiliation and social recognition (Kaidanovich-Beilin et al, 2011): the testing occurred in a plastic three-chamber neutral box cage (plexiglas, 60 cm length, 40 cm width, and 22 cm height). Each mouse was allowed to explore freely for 5 min in the cage to habituate to the testing conditions (adaptation). During the social affiliation session, a new mouse (Stranger 1) was placed in a wire containment cup that was located in one side chamber. Subject mouse was allowed free access to explore each of the three chambers for 10 min. During the social recognition session, a second new mouse (Stranger 2) placed to a wire containment cup was put in the opposite side chamber. The subject mouse freely explored each of the three chambers for 10 min during two sections. The time spent in social interaction (sniffing) was recorded. (6) Nest building test: the procedure and rating scale were performed as literature (Deacon, 2006). Briefly, the test used pressed cotton wafers as nestlet, transfer the mice to individual testing cage 1 h before the dark phase, place one nestlet in each cage but no other environmental enrichment items. Assess the nests the next morning on a rating scale of 1–5 based on literature (Deacon, 2006).

### Statistics and reproducibility

All measured data were presented as mean ± SEM. Sample sizes of mouse neurobehavioral experiments were designed according to our published studies or literature. All cell biology and molecular biology experiments were repeated independently at least twice. Two-tailed unpaired *t* test was used for analyses which involved only two groups for comparison, and ANOVA and appropriate post hoc tests were used for analyses which involved more than two groups for comparisons. Software for performing statistics included Prism and Statistical Package for the Social Sciences software (SPSS), and *P*-value of less than 0.05 was considered statistically significant.

## Supplementary Material

Reviewer comments
