# Supplementary material for: Brain is an endocrine organ through secretion and nuclear transfer of parathymosin
Source: Life Sci Alliance. 2020 Oct 21;3(12):e202000917. doi: 10.26508/lsa.202000917 (PMC7652378; doi:10.26508/lsa.202000917)
Supplement: Reviewer comments [file LSA-2020-00917_review_history.pdf]

Life Science Alliance

Brain is an endocrine organ through secretion and nuclear transfer of parathymosin

Bin Yu, Yizhe Tang, and Dongsheng Cai
DOI: https://doi.org/10.26508/lsa.202000917

Corresponding author(s): Dongsheng Cai, Albert Einstein College of Medicine, New York

Review Timeline:

Submission Date:	2020-09-25
Editorial Decision:	2020-09-28
Revision Received:	2020-10-01
Editorial Decision:	2020-10-02
Revision Received:	2020-10-04
Accepted:	2020-10-05

Scientific Editor: Shachi Bhatt

Transaction Report:

(Note: With the exception of the correction of typographical or spelling errors that could be a source of ambiguity, letters and reports are not edited. The original formatting of letters and referee reports may not be reflected in this compilation.)

No full Peer Review Process File is available with this article, as the authors have chosen not to make the review process public in this case

Please note that the manuscript was previously reviewed at another journal and the reports were taken into account in the decision-making process at Life Science Alliance.

September 28, 2020

Re: Life Science Alliance manuscript #LSA-2020-00917-T

Dr. Dongsheng Cai
Albert Einstein College of Medicine, New York
Department of Molelcular Pharmacology
1300 Morris Park Avenue
Forchheimer 216A
Bronx, NY 10461

Dear Dr. Cai,

Thank you for transferring your manuscript entitled "Secretion and nuclear transfer of PTMS in the brain and its neurobehavioral protection" to Life Science Alliance (LSA)/

Just for a brief overview, the paper was reviewed at a partner journal, where the reviewers thought that the findings were interesting, but lacking in molecular mechanistic depth. Given the novel findings related to the transfer of PTMS from htNSCs to other cells and its effect on aging, the manuscript was considered to be appropriate for publication in LSA with the following minor edits:

+ Text changes / Clarifications and Discussion points
++ Please address Reviewer 1's pts 1-4, 7 -9, and 11- 16; Reviewer 2's general pts 1 and 3, and specific pts 1-4 (pg 7, 8, 9, 10) and 6-7 (pg 12, 13); Reviewer 3's pts 1-4 and 6 with text changes - additional discussion, clarifications and quantification

+ If readily available, we would appreciate if you can provide data in response to Reviewer 1's pt 6 and 17; Reviewer 2's pts 4 and 10; and Reviewer 3's pt 5 and 8. If these data are not readily available, we request you to tone down the respective conclusions

+ We would appreciate if you can provide us antibody specificity data as requested by Reviewer 1, pt 5; promoter expression control data as requested by Reviewer 1, pt 10

+ The authors have personally communicated with the LSA editor that the concern about image quality for figure 3c, 3b and 7b, 7c (Reviewer 3, pt 7) is a result of unclear writing, and can be satisfied through text changes.

+ Along with the above changes, LSA would also request the authors to submit a point-by-point rebuttal to all the concerns raised by the referees with the revised manuscript.

To upload the revised version of your manuscript, please log in to your account:
https://lsa.msubmit.net/cgi-bin/main.plex
You will be guided to complete the submission of your revised manuscript and to fill in all necessary information. Please get in touch in case you do not know or remember your login name.

We would be happy to discuss the individual revision points further with you should this be helpful. While you are revising your manuscript, please also attend to the below editorial points to help expedite the publication of your manuscript. Please direct any editorial questions to the journal office.

When submitting the revision, please include a letter addressing the reviewers' comments point by point.

Thank you for considering Life Science Alliance (LSA) as an appropriate venue for your research. We look forward to receiving your revised manuscript.

Sincerely,

Shachi Bhatt, Ph.D.
Executive Editor
Life Science Alliance

A. THESE ITEMS ARE REQUIRED FOR REVISIONS

-- A letter addressing the reviewers' comments point by point.

-- An editable version of the final text (.DOC or .DOCX) is needed for copyediting (no PDFs).

-- High-resolution figure, supplementary figure and video files uploaded as individual files: See our detailed guidelines for preparing your production-ready images, https://www.life-science-alliance.org/authors

-- Summary blurb (enter in submission system): A short text summarizing in a single sentence the study (max. 200 characters including spaces). This text is used in conjunction with the titles of papers, hence should be informative and complementary to the title and running title. It should describe the context and significance of the findings for a general readership; it should be written in the present tense and refer to the work in the third person. Author names should not be mentioned.

B. MANUSCRIPT ORGANIZATION AND FORMATTING:

Full guidelines are available on our Instructions for Authors page, https://www.life-science-alliance.org/authors

We encourage our authors to provide original source data, particularly uncropped/-processed electrophoretic blots and spreadsheets for the main figures of the manuscript. If you would like to add source data, we would welcome one PDF/Excel-file per figure for this information. These files will be linked online as supplementary "Source Data" files.

IMPORTANT: It is Life Science Alliance policy that if requested, original data images must be made available. Failure to provide original images upon request will result in unavoidable delays in publication. Please ensure that you have access to all original microscopy and blot data images before submitting your revision.

October 2, 2020

RE: Life Science Alliance Manuscript #LSA-2020-00917-TR

Dr. Dongsheng Cai
Albert Einstein College of Medicine, New York
Department of Molelcular Pharmacology
1300 Morris Park Avenue
Forchheimer 216A
Bronx, NY 10461

Dear Dr. Cai,

Thank you for submitting your revised manuscript entitled "Brain is an endocrine organ through secretion and nuclear transfer of parathymosin". We would be happy to publish your paper in Life Science Alliance pending final revisions necessary to meet our formatting guidelines.

Along with the points mentioned below, please also address to the following:
-please upload both your main figures and your supplementary figures as single files
-please add your supplementary figure legends to the main manuscript text (directly under your main figure legends)
-the figure callouts are all lower case (a,b,c, etc.) - please change them to upper case (A, B, C, etc..) in the legends as well as in the manuscript text

If you are planning a press release on your work, please inform us immediately to allow informing our production team and scheduling a release date.

To upload the final version of your manuscript, please log in to your account:
https://lsa.msubmit.net/cgi-bin/main.plex
You will be guided to complete the submission of your revised manuscript and to fill in all necessary information. Please get in touch in case you do not know or remember your login name.

To avoid unnecessary delays in the acceptance and publication of your paper, please read the following information carefully.

A. FINAL FILES:

These items are required for acceptance.

-- An editable version of the final text (.DOC or .DOCX) is needed for copyediting (no PDFs).

-- High-resolution figure, supplementary figure and video files uploaded as individual files: See our detailed guidelines for preparing your production-ready images, https://www.life-science-alliance.org/authors

-- Summary blurb (enter in submission system): A short text summarizing in a single sentence the study (max. 200 characters including spaces). This text is used in conjunction with the titles of

papers, hence should be informative and complementary to the title. It should describe the context and significance of the findings for a general readership; it should be written in the present tense and refer to the work in the third person. Author names should not be mentioned.

B. MANUSCRIPT ORGANIZATION AND FORMATTING:

Full guidelines are available on our Instructions for Authors page, https://www.life-science-alliance.org/authors

We encourage our authors to provide original source data, particularly uncropped/-processed electrophoretic blots and spreadsheets for the main figures of the manuscript. If you would like to add source data, we would welcome one PDF/Excel-file per figure for this information. These files will be linked online as supplementary "Source Data" files.

Submission of a paper that does not conform to Life Science Alliance guidelines will delay the acceptance of your manuscript.

It is Life Science Alliance policy that if requested, original data images must be made available to the editors. Failure to provide original images upon request will result in unavoidable delays in publication. Please ensure that you have access to all original data images prior to final submission.

The license to publish form must be signed before your manuscript can be sent to production. A link to the electronic license to publish form will be sent to the corresponding author only. Please take a moment to check your funder requirements.

Reviews, decision letters, and point-by-point responses associated with peer-review at Life Science Alliance will be published online, alongside the manuscript. If you do want to opt out of having the reviewer reports and your point-by-point responses displayed, please let us know immediately.

Thank you for your attention to these final processing requirements. Please revise and format the manuscript and upload materials within 7 days.

Thank you for this interesting contribution, we look forward to publishing your paper in Life Science Alliance.

Sincerely,

Shachi Bhatt, Ph.D.
Executive Editor
Life Science Alliance
https://www.life-science-alliance.org/
Tweet @SciBhatt @LSAjournal

October 5, 2020

RE: Life Science Alliance Manuscript #LSA-2020-00917-TRR

Dr. Dongsheng Cai
Albert Einstein College of Medicine, New York
Department of Molelcular Pharmacology
1300 Morris Park Avenue
Forchheimer 216A
Bronx, NY 10461

Dear Dr. Cai,

Thank you for submitting your Research Article entitled "Brain is an endocrine organ through secretion and nuclear transfer of parathymosin". It is a pleasure to let you know that your manuscript is now accepted for publication in Life Science Alliance. Congratulations on this interesting work.

The final published version of your manuscript will be deposited by us to PubMed Central upon online publication.

Your manuscript will now progress through copyediting and proofing. It is journal policy that authors provide original data upon request.

Reviews, decision letters, and point-by-point responses associated with peer-review at Life Science Alliance will be published online, alongside the manuscript. If you do want to opt out of having the reviewer reports and your point-by-point responses displayed, please let us know immediately.

IMPORTANT: If you will be unreachable at any time, please provide us with the email address of an alternate author. Failure to respond to routine queries may lead to unavoidable delays in publication.

Scheduling details will be available from our production department. You will receive proofs shortly before the publication date. Only essential corrections can be made at the proof stage so if there are any minor final changes you wish to make to the manuscript, please let the journal office know now.

DISTRIBUTION OF MATERIALS:
Authors are required to distribute freely any materials used in experiments published in Life Science Alliance. Authors are encouraged to deposit materials used in their studies to the appropriate repositories for distribution to researchers.

You can contact the journal office with any questions, contact@life-science-alliance.org

Again, congratulations on a very nice paper. I hope you found the review process to be constructive and are pleased with how the manuscript was handled editorially. We look forward to future exciting submissions from your lab.

Sincerely,

Shachi Bhatt, Ph.D.
Executive Editor
Life Science Alliance
https://www.life-science-alliance.org/
Tweet @SciBhatt @LSAjournal
